# New Insights into Profibrotic Myofibroblast Formation in Systemic Sclerosis: When the Vascular Wall Becomes the Enemy

**DOI:** 10.3390/life11070610

**Published:** 2021-06-24

**Authors:** Eloisa Romano, Irene Rosa, Bianca Saveria Fioretto, Marco Matucci-Cerinic, Mirko Manetti

**Affiliations:** 1 Department of Experimental and Clinical Medicine, Division of Rheumatology, University of Florence, 50134 Florence, Italy; eloisa.romano@unifi.it (E.R.); biancafioretto@icloud.com (B.S.F.); marco.matuccicerinic@unifi.it (M.M.-C.); 2 Department of Experimental and Clinical Medicine, Section of Anatomy and Histology, University of Florence, 50134 Florence, Italy; irene.rosa@unifi.it

**Keywords:** systemic sclerosis, fibrosis, myofibroblasts, endothelial-to-mesenchymal transition, pericytes, vascular smooth muscle cells, vasculopathy

## Abstract

In systemic sclerosis (SSc), abnormalities in microvessel morphology occur early and evolve into a distinctive vasculopathy that relentlessly advances in parallel with the development of tissue fibrosis orchestrated by myofibroblasts in nearly all affected organs. Our knowledge of the cellular and molecular mechanisms underlying such a unique relationship between SSc-related vasculopathy and fibrosis has profoundly changed over the last few years. Indeed, increasing evidence has suggested that endothelial-to-mesenchymal transition (EndoMT), a process in which profibrotic myofibroblasts originate from endothelial cells, may take center stage in SSc pathogenesis. While in arterioles and small arteries EndoMT may lead to the accumulation of myofibroblasts within the vessel wall and development of fibroproliferative vascular lesions, in capillary vessels it may instead result in vascular destruction and formation of myofibroblasts that migrate into the perivascular space with consequent tissue fibrosis and microvessel rarefaction, which are hallmarks of SSc. Besides endothelial cells, other vascular wall-resident cells, such as pericytes and vascular smooth muscle cells, may acquire a myofibroblast-like synthetic phenotype contributing to both SSc-related vascular dysfunction and fibrosis. A deeper understanding of the mechanisms underlying the differentiation of myofibroblasts inside the vessel wall provides the rationale for novel targeted therapeutic strategies for the treatment of SSc.

## 1. Introduction

Systemic sclerosis (SSc, or scleroderma) is a multifaceted autoimmune disorder characterized by widespread microvascular abnormalities occurring early during the disease and evolving into a distinctive vasculopathy that inexorably advances in parallel with the development of tissue fibrosis [1,2,3,4]. The microvascular disease may present clinically as Raynaud’s phenomenon, abnormal nailfold capillaries, digital ulcers, pulmonary arterial hypertension (PAH) and scleroderma renal crisis [5,6] and together with myofibroblast-orchestrated untreatable skin and internal organ fibrosis, often lead to organ failure accounting for the high morbidity and mortality of SSc [1,2,3]. Over the past few years, many steps forward have been made in the knowledge of the cellular and molecular mechanisms underlying the unique relationship between SSc-related vasculopathy and fibrosis [2,3,7]. In this context, a process in which profibrotic myofibroblasts originate from vascular endothelial cells (ECs), namely endothelial-to-myofibroblast transition, commonly referred to as endothelial-to-mesenchymal transition (EndoMT), has been assumed to take center stage [8,9]. Indeed, if in arterioles and small arteries EndoMT is supposed to lead to the accumulation of myofibroblasts within the vessel wall, with consequent development of fibroproliferative vascular lesions, in capillary vessels it may result in significant microvascular destruction due to disappearance of ECs and concomitant formation of myofibroblasts migrating into the perivascular space, thus leading to tissue fibrosis and microvessel rarefaction [8]. Interestingly, it appears that other vascular wall-resident cells, such as pericytes and vascular smooth muscle cells (VSMCs), may acquire a myofibroblast-like profibrotic phenotype contributing to both SSc-related vascular dysfunction and fibrosis [8].

In this review, after a brief description of the main pathologic characteristics of the microvasculature in SSc, we discuss the mechanisms underlying the differentiation of myofibroblasts inside the vessel wall and their contribution to vasculopathy and tissue fibrosis in this disease. An overview of the main therapeutic strategies that have been proposed to counteract SSc-associated tissue fibrosis, with a particular focus on the blockade of profibrotic myofibroblast differentiation from vascular wall-resident cells, will also be provided.

## 2. The Normal Vascular Wall

The vascular system is fundamental to deliver oxygen and nutrients to tissues and remove CO_2_ and tissue waste matter, a process that is conducted by both macro- and micro-vasculature. The macro-vasculature is composed of large vessels that rapidly transport blood toward or away from organs, while micro-vasculature consists of small vessels including arterioles, capillaries and postcapillary venules [10,11]. The innermost layer of each of these vessels is lined with ECs that interact with the circulation and respond to various stimuli, coordinating the vessel response to changes in nutrients, oxygen and different molecules including hormones. Although all blood vascular ECs are thought to derive from mesodermal progenitors, it has been demonstrated that they are able not only to express different genes/proteins depending on the type of vessel to which they belong, but also to acquire tissue-specific characteristics, especially in capillaries [10,11]. As an example, ECs comprising brain and retina capillaries express high levels of tight junctions in order to restrict the passage of different molecules in the circulation, while in liver and kidney capillaries, these cells present fenestrations that allow for extensive filtration of a variety of factors [10]. Inside the vascular wall, ECs are also surrounded by other cell types such as pericytes, VSMCs and fibroblasts/fibroblast-like cells, in a measure that varies depending upon anatomical location within the vasculature, as well as organ or tissue type [11]. Structurally, the wall of arteries and arterioles consists of three layers, the innermost and thinnest of which is represented by the tunica intima, a single continuous EC layer supported by subendothelial connective tissue. The tunica media surrounds the tunica intima and is comprised of VSMCs that control the vessel caliber and the volume of blood flow via contraction or relaxation, as well as elastic and collagen fibers circularly arranged around the vessel; the tunica adventitia instead constitutes the outermost layer and is entirely composed of connective tissue that allows to anchor vessels to surrounding tissues [11,12]. In particular, arteries present a thick vessel wall with multiple layers of VSMCs, while arterioles are surrounded by a thinner muscular wall [11]. As far as the structural organization of small capillaries, their thin wall presents a single layer of ECs connected with a basement membrane hosting pericytes, a kind of supportive cells that maintain capillary vessel integrity [10,13,14]. Moreover, because of the absence of a continuous smooth muscle layer, the wall of these microvessels lacks vasomotor function [11]. The structure of capillaries is organ-specific, with a morphology that is perfectly suited for the needs of the tissue [10,15].

## 3. Vascular Wall Alterations in Systemic Sclerosis and Related Clinical Manifestations

As previously mentioned, microvascular abnormalities are the earliest manifestations of SSc and typically precede immune dysregulation and tissue fibrosis. Toxic, autoimmune, metabolic or infectious agents may severely damage the endothelium, thus triggering a chronic injury that causes progressive and permanent alterations affecting ECs, which may undergo activation or apoptosis, as well as VSMCs and pericytes [7,8,16,17]. Such a vascular damage can determine intimal proliferation, with consequent luminal narrowing finally leading to obliteration of small arteries and arterioles, as well as capillary dysfunction and necrosis, all microvascular changes that in the peripheral circulation are clinically mirrored by Raynaud’s phenomenon. The pathologic changes in the microvascular tree are not limited to the skin, but may also affect internal organs such as lungs, heart, kidneys, and the gastrointestinal tract. In particular, distorted and irregular capillary loops can be detected in the capillary network of all involved organs, reflecting the diffuse nature of SSc-associated vascular disorder, even in sites not affected by tissue fibrosis [7,17,18].

### 3.1. Skin

SSc-related vascular abnormalities are particularly evident in the skin, where they can be easily observed by means of nailfold video-capillaroscopy and typically affect the microcirculation, namely capillaries and small blood vessels [6,19]. In particular, affected arterioles and small arteries are characterized by fibrosis of the tunica adventitia, hyperplasia of the tunica media, swelling and thickening of the tunica intima and luminal occlusion, while capillary alterations range from microhemorrhages and giant or distorted capillaries to capillary rarefaction and loss [7,18,20]. Moreover, another pathologic feature of the affected capillaries is represented by the so-called “vascular leak” due to the opening of EC tight junctions and the consequent perivascular mononuclear cell infiltration and tissue edema [20]. From a clinical point of view, scleroderma-associated peripheral microvascular disease manifests as an uncontrolled recurrent digital vasospasm also known as Raynaud’s phenomenon, edematous puffy fingers, telangiectasias that are histologically identified as dilated blood vessels in the superficial dermis, fingertip ulceration reflecting tissue ischemia due to underlying vasculopathy and an abnormal procoagulant state with thrombosis and fibrin deposition [1,2,3,7,20].

### 3.2. Lung

Lung vascular damage is mainly represented by PAH, a condition that plays an important role in SSc prognosis, as it is related to increased mortality [4,21,22,23]. Histologically, early pathologic lesions of PAH manifest with medial and intimal thickening, subsequent subintimal fibrosis in an onion-skin pattern and eventual reduplication of the internal elastic lamina [24], while later lesions comprise plexiform and “glomeruloid” nodules in small arteries with fibrin thrombi formation [24]. Acute necrotizing arteritis with fibrinoid necrosis can also rarely be seen. Of note, patients with SSc-associated PAH are characterized by a worse prognosis in respect to patients with idiopathic PAH [25], an outcome that has been linked to the additional presence of veno-occlusive disease, with fibrosis and intimal proliferation of preseptal venules and veins, in such patients [21,26].

### 3.3. Kidney

Scleroderma renal crisis is another severe complication of SSc that occurs in up to 15% of patients and is associated with a vasculopathy of arcuate and interlobular renal arteries resulting in increased activity of the renin-angiotensin system, thus leading to severe hypertension followed by acute renal failure [4,7,27,28,29]. Affected kidneys are macroscopically characterized by multiple surface petechial hemorrhages and wedge-shaped cortical infarcts, while from a histological point of view, they present different lesions according to the stage of the disease. In particular, accumulation of myxoid material in the intima of interlobular arteries and fibrinoid necrosis of afferent arterioles are evident in early disease, whereas vessel intimal proliferation in an onion-skin pattern and glomerular ischemic changes with collapse and wall thickening are present in later stages [24,27,28,29]. Interestingly, vascular fibrosis and accumulation of extracellular matrix (ECM) material is histologically evident also in patients who never demonstrated clinical symptoms of scleroderma renal crisis [24,30].

### 3.4. Heart

Cardiovascular disease is the cause of death in 14–36% of all SSc cases with a wide range of pathologic manifestations including microvascular disease, atherosclerotic coronary artery disease, myocardial fibrosis and pericarditis [31,32]. Microvascular disease manifests with nonocclusive concentric intimal hyperplasia of myocardial arterioles and unique myocardial lesions known as “contraction band necrosis”, which is due to a sort of cardiac Raynaud’s phenomenon, i.e., a sequelae of perfusion and reperfusion injuries (microvascular coronary vasospasm) resulting in ischemic events finally leading to arrhythmias and cardiac dysfunction [4,7,33,34]. Moreover, different vascular abnormalities such as fibrinoid necrosis, mural fibrosis, intimal proliferation and medial hyperplasia have been detected in in coronary arteries of <1 mm in size [24]. Strikingly, intermittent vascular spasm, reperfusion injury and ischemic necrosis are considered to play a pivotal role in inducing myocardial fibrosis, histologically manifesting as a patchy replacement of cardiac myocytes with acellular collagenous material throughout the myocardium [4,24,33,34]. As far as macrovascular involvement is concerned, arterial intimal thickening and occlusion by atheromatous plaques characterized by a plenty of foamy macrophages and cholesterol clefts with an overlying fibrous cap have been demonstrated in some SSc patients [6,35,36,37]. Of note, a significant association between aortic root dilation and impairment of capillary density at nailfold video-capillaroscopy was recently detected in a multicenter cohort of SSc patients, suggesting that SSc-related peripheral microangiopathy could mimic that of aortic vasa vasorum [6].

### 3.5. Gastrointestinal Tract

Gastrointestinal (GI) complications including gastroesophageal reflux, mucosal ulceration, malabsorption, delayed gastric emptying, and constipation affect more than 90% of SSc patients and strongly contribute to a decreased quality of life [38,39,40,41]. As in other organs, although the pathognomonic lesion of SSc GI tract is represented mainly by fibrosis of the muscular layers, vasculopathy is thought to be the underlying cause of such fibrotic process, as intimal thickening of the muscularis propria vessels is commonly observed [24,42]. Vascular abnormalities are also evident within the GI mucosa, with 15% of SSc patients experiencing GI hemorrhages, mainly due to the presence of telangiectasias, i.e., dilated blood vessels in the lamina propria, similar to those seen in the skin [24]. Another possible source of GI bleeding, even if less frequent, may be represented by gastric antral vascular ectasia, a condition that is commonly known as “watermelon stomach” because of the presence of dilated blood vessels in the antrum that appear as parallel red stripes [24].

## 4. The Pathogenic Role of Vascular Wall-Resident Cells in Systemic Sclerosis: Linking Vasculopathy to Fibrosis

Since microvascular injury clearly represents the initial event in SSc pathogenesis, but the true remarkable feature of the disease is represented by widespread tissue fibrosis, it is conceivable that different vascular wall-resident cells including ECs, pericytes and VSMCs may be central in establishing this unique relationship between vasculopathy and fibrosis.

### 4.1. Endothelial Cells

In recent years, numerous studies have significantly expanded our knowledge about the plausible mechanisms underlying EC dysregulation and the contribution of these cells to the main pathogenic features of SSc. The initial EC damage may be induced by different triggers including anti-endothelial autoantibodies, infections, environmental factors, ischemia-reperfusion events and reactive oxygen species [1]. Once injured, ECs are believed to undergo two distinct fates: cell activation or cell death [4,19]. In the first case, ECs function abnormally, becoming unable to promote angiogenesis and leading to increased endothelin-1 (ET-1) production and impaired nitric oxide and prostacyclin release, an imbalance that mediates vasospasm and contributes to both intimal proliferation and vascular wall fibrosis [2,3,4,18]. Activated ECs in SSc also undergo cytoskeletal rearrangement, loss of tight junctions and increase in adhesion molecule, cytokine and growth factor expression, thus enhancing the interaction with circulating immune cells and contributing to tissue inflammation [2,3,4,18]. Moreover, activated ECs may determine platelet activation and consequent intravascular fibrin deposition, finally resulting in luminal narrowing and vessel obstruction [2,3,4,18]. On the other hand, EC apoptosis is known to account for the loss of peripheral capillary network and the consequent chronic tissue ischemia, which cannot be compensated by an adequate and functional angiogenesis [2,3,18].

In this context, the recently described EndoMT, a transdifferentiation process consisting of a phenotypic switch of ECs toward profibrotic myofibroblasts, adds to endothelial activation/loss in promoting both SSc-related microvascular dysfunction and tissue fibrosis [8,9,43]. In particular, during EndoMT, ECs go through a profound morphologic change consisting of disaggregation, loss of polarity, acquisition of the typical fibroblast-like spindly shape, migratory and invasive abilities and enhanced resistance to apoptosis [8,43,44,45]. Immunophenotypically, ECs encounter a downregulation of their specific markers CD31/platelet-endothelial cell adhesion molecule-1, vascular endothelial (VE)-cadherin and von Willebrand factor (vWF) and acquire myofibroblast products, including α-smooth muscle actin (α-SMA), S100A4/fibroblast-specific protein-1 and type I collagen [8,43,44,45]. A pivotal role in inducing the gene expression program responsible for EndoMT is played by the activation, stabilization and nuclear translocation of Snail1, a transcription factor that is upregulated in SSc [8,43,44,45]. Furthermore, the EndoMT process may be triggered by several cytokines and growth factors such as transforming growth factor-β (TGF-β), ET-1, interleukin-1β (IL1-β), tumor necrosis factor-α (TNF-α), Notch and Wnt ligands, as well as by caveolin-1 deficiency, hypoxia and oxidative stress [8,9,43,44].

In recent years, increasing evidence supports the implication of EndoMT in the development of the main pathologic aspects of SSc, namely dermal fibrosis, interstitial lung disease (ILD) and PAH [8,43,44,46,47,48].

**Figure 1:**
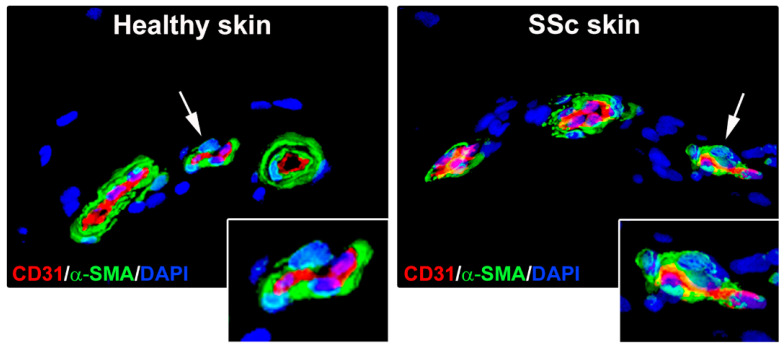
Microphotographs of skin sections from healthy individuals and patients with systemic sclerosis (SSc) subjected to double immunofluorescence for the endothelial cell marker CD31 (red) and the myofibroblast marker α-smooth muscle actin (α-SMA; green). Nuclei were counterstained with 4′,6-diamidino-2-phenylindole (DAPI; blue). In healthy skin microvessels, α-SMA expression is restricted to pericytes and vascular smooth muscle cells surrounding endothelial cells. In SSc skin microvessels, endothelial cells undergoing endothelial-to-myofibroblast transition display colocalization of CD31 and α-SMA (yellow staining). In both panels, the inset represents a higher magnification view of the dermal microvessel pointed by arrow.

In the skin, the concomitant expression of endothelial and myofibroblast markers has been detected in ECs from SSc-affected dermal microvessels (Figure 1) and in both the bleomycin-induced and the urokinase-type plasminogen activator receptor (uPAR)-deficient mouse models of SSc [8,9,43,46,49]. Moreover, explanted SSc dermal microvascular ECs were found not only to co-express endothelial and myofibroblast markers, but also to exhibit a spindle-shaped morphology and a contractile phenotype [46].

Of note, comparable phenotypic, morphologic and functional features were also detected in healthy dermal microvascular ECs upon stimulation with SSc sera [46]. In this regard, the pro-EndoMT effect exerted by SSc sera has been in part attributed to matrix metalloproteinase-12 (MMP-12)-dependent uPAR cleavage, a process that had already been implicated in both SSc impaired angiogenesis and fibroblast-to-myofibroblast differentiation [46]. Strikingly, MMP-12 is known to be significantly increased in SSc sera and tissues [50] and its upregulation has been recently supposed to be partially triggered by SSc fibroblast-mediated extracellular acidosis [51]. Indeed, the acidic milieu generated by the highly glycolytic SSc fibroblasts has been reported to induce MMP-12 overexpression and consequent uPAR cleavage in ECs, making these cells prone to undergo EndoMT [51]. Recently, it has also been demonstrated that SSc sera are able to induce a fibroblastic morphology in murine ECs. In particular, sera from Scl70 + SSc patients significantly upregulated the expression of the EndoMT markers plasminogen activator inhibitor-1, type I collagen, and α-SMA in respect to control sera, suggesting that EndoMT-promoting signals are more represented in those patients characterized by a higher risk of visceral involvement [52]. Two different studies have further demonstrated the role of the profibrotic mediators ET-1 and TGF-β in the induction of the EndoMT program in dermal ECs. In the former, ET-1 and TGF-β stimulation was able to trigger EndoMT in SSc dermal microvascular ECs [53], while in the latter, microvascular ECs explanted from the unaffected skin of SSc patients were reported to transdifferentiate toward profibrotic myofibroblasts when co-cultured with SSc fibroblasts from affected skin and concomitantly treated with ET-1 and TGF-β [54]. Besides triggering EndoMT in the skin, the master fibrogenic cytokine TGF-β is able to induce this transdifferentiation process also in internal organs. Experimentally, constitutive EC-specific activation of TGF-β signaling in transgenic mice indeed led to a strong expression of genes related to myofibroblast activation, resulting in a severe cutaneous and pulmonary fibrosis, with important perivascular ECM deposition and subendothelial thickening of small arterioles resembling the typical SSc alterations [55]. Among the various mechanisms of action exerted by TGF-β in inducing EndoMT, it has been demonstrated that this profibrotic molecule is able to abrogate the activity of Friend leukemia integration factor 1 (Fli1), a transcription factor important for the maintenance of EC homeostasis [8,43]. Accordingly, SSc dermal microvascular ECs undergoing EndoMT showed a significant Fli1 downregulation in culture [46] while ex vivo, mice with a conditional Fli1 deletion in ECs exhibited a significant downregulation of EC markers together with several vascular dysfunctions consistent with those detected in SSc microvasculature [56]. Moreover, Fli1 haploinsufficiency has been found to induce a profibrotic phenotype in dermal ECs of bleomycin-treated mice, further strengthening the involvement of this transcription factor in EndoMT during the development of cutaneous fibrosis [57,58]. Recently, dermal ECs explanted from KLF5+/−; Fli1+/− mice, a new animal model resembling the fundamental pathologic features of SSc, were reported not only to be defective in performing in vitro angiogenesis, but also to have a reduced expression of VE-cadherin and CD31, suggesting the occurrence of EndoMT, as observed in SSc-affected ECs, and highlighting that a deficiency of KLF5 transcription factor may be another important trigger of this process [59]. SSc-related EndoMT has been recently found to be induced also by oncostatin M, a member of the IL-6 family, and the inflammatory lipid mediator leukotriene B4, whose expression levels are increased in SSc [60,61]. In particular, oncostatin M was able to induce EndoMT-like morphologic changes in healthy dermal microvascular ECs, while leukotriene B4 promoted EndoMT in human umbilical vein ECs via the phosphatidylinositol 3-kinase (PI3K)/protein kinase B (AKT)/mammalian target of the rapamycin (mTOR) pathway, independently of TGF-β1 release [61]. A putative contribution in EndoMT induction has additionally been attributed, even if only in experimental scleroderma, to abnormal fibrillin-1 expression and chronic oxidative stress in the tight-skin mouse model of SSc [62] and to interferon regulatory factor 5 (IRF5), as demonstrated by the fact that EndoMT is inhibited in the IRF5 knockout mouse model [63]. Interestingly, some molecules that are known to have a role in both SSc-related impaired angiogenesis and tissue fibrosis, namely vascular endothelial growth factor (VEGF)165b, soluble α-Klotho and caveolin-1 [64,65,66,67], have been correlated to the induction of EndoMT as well [54,68,69,70]. As an example, the antiangiogenic factor VEGF165b has been found to be significantly overexpressed in transitioning ECs of an in vitro model of EndoMT consisting of healthy dermal microvascular ECs cocultured with SSc fibroblasts and concomitantly stimulated with ET-1 and TGF-β [54]. Furthermore, among the molecules that have been recently studied in SSc, it is worth mentioning soluble α-Klotho, a protein with vasculoprotective effects whose expression has been found to be decreased in SSc microvascular ECs and whose in vitro administration was able to significantly improve endothelial functions by acting as a powerful proangiogenic factor [65]. Interestingly, this pleiotropic molecule was shown to significantly ameliorate EndoMT progression in a mouse model of renal fibrosis by targeting TGF-β1/Smad/Snail1 signaling, evidence suggesting a possible therapeutic use of α-Klotho against pathologic fibrosis [68]. As far as the caveolae-associated protein caveolin-1 is concerned, it acts as a crucial inhibitor of tissue fibrosis and its downregulation in different SSc dermal cell types including microvascular ECs and fibroblasts has been broadly implicated in SSc-related tissue fibrosis [66,67,71]. Remarkably, caveolin-1 deficiency has also been related to EndoMT, with pulmonary ECs explanted from caveolin-1 knockout mice displaying high levels of mesenchymal/myofibroblast markers such as type I collagen, α-SMA and Snail [70]. Finally, the antiangiogenic molecule semaphorin 3E, which has been found to contribute to defective angiogenesis of SSc dermal microvascular ECs and to participate in epithelial-to-mesenchymal transition, a transdifferentiation process similar to EndoMT, might have a possible role in SSc-associated EndoMT, as well [8,43,72].

The presence of ECs undergoing EndoMT has been detected not only in the skin but also in the lungs of SSc patients, where this cell transdifferentiation may contribute to two important SSc-related complications such as PAH and ILD [47,48,73,74]. Colocalization of vWF and α-SMA, indicative of the occurrence of EndoMT, has been reported in both pulmonary arterioles of patients with SSc-associated PAH and in a murine model of hypoxia-induced PAH [75]. Moreover, the same authors reported that the exposure of healthy pulmonary artery ECs to inflammatory cytokines led to a significant reorganization of their actin cytoskeleton and the development of a myofibroblast-like morphology [75]. Interestingly, ECs in intermediate stages of EndoMT have been also found in lung tissue of patients suffering from SSc-associated ILD [74]. This evidence has been further confirmed in a subsequent study reporting significant expression of different mesenchymal cell/myofibroblast-specific genes in lung microvascular ECs isolated from SSc patients affected by ILD [76]. Recently, the fate of ECs was investigated during bleomycin-induced pulmonary fibrosis by means of EC-specific genetic lineage tracing mice. Here, the induction of fibrosis caused an increased expression of different myofibroblast markers, but no changes in endothelial markers in lung ECs, suggesting the occurrence of a partial EndoMT [52]. Furthermore, it was demonstrated that macrophage depletion, together with bleomycin injection, was able to contribute to EndoMT-associated gene increase in pulmonary ECs, indicating a possible role of lung macrophages in preserving EC morphology and preventing fibrosis [52].

**Figure 2:**
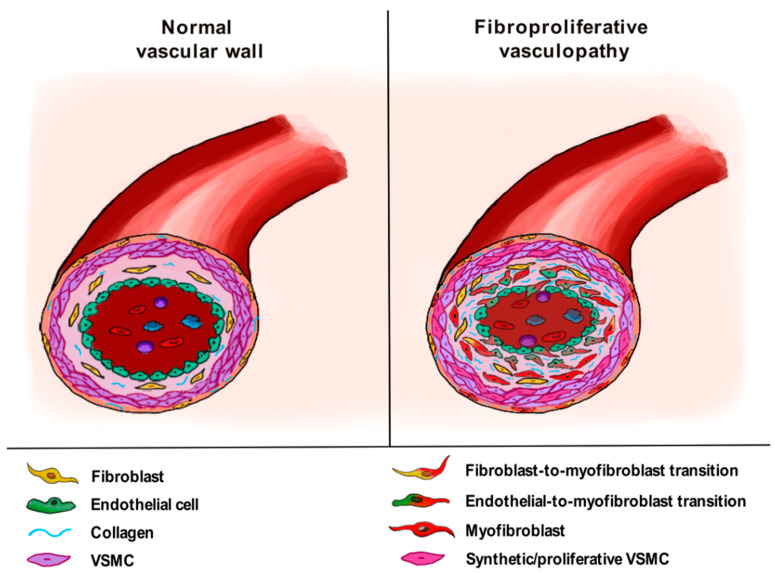
In systemic sclerosis, vasculopathy affecting arterioles and small arteries in the skin and internal organs features fibrosis of the tunica adventitia, hyperplasia of the tunica media and thickening of the tunica intima, eventually leading to luminal occlusion. Fibroproliferative structural changes of the vascular wall are driven mainly by the differentiation of profibrotic myofibroblasts from fibroblasts and endothelial cells through the processes of fibroblast-to-myofibroblast transition and endothelial-to-myofibroblast transition, respectively, as well as the transition of vascular smooth muscle cells (VSMCs) from a contractile to a synthetic/proliferative phenotype.

As recently proposed, it is important to point out that the SSc-related EndoMT process may play different pathogenic roles on the basis of the type of affected vessels, both in the skin and in internal organs [8,43,47]. In particular, when affecting arterioles and small arteries, EndoMT may contribute to the so-called “fibroproliferative vasculopathy”, consisting of myofibroblast accumulation in the vessel subintima and media, with consequent thickening of the vessel wall and occlusive vascular disease (Figure 2). The main clinical manifestations of such a profound vessel remodeling are represented by digital ulcers, gangrene of the extremities, SSc-associated PAH, scleroderma renal crisis and myocardial blood vessel anomalies [8,43,47].

**Figure 3:**
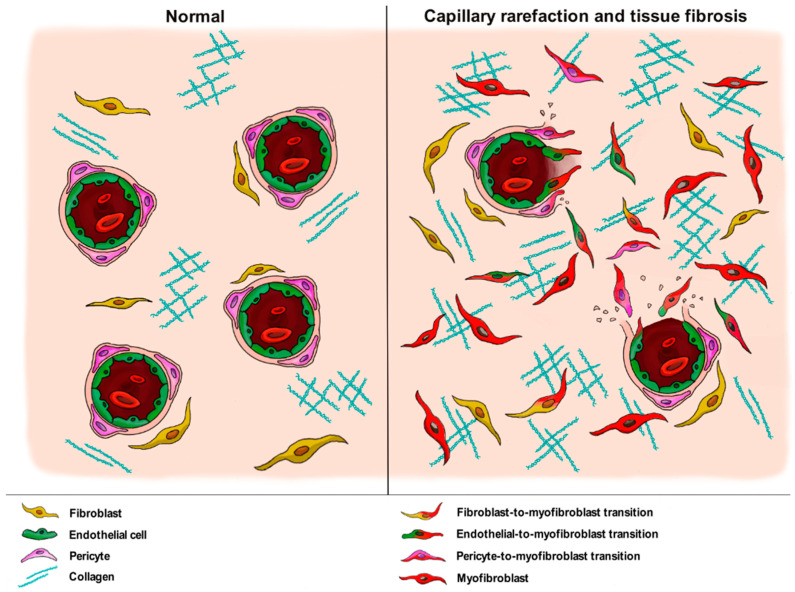
In the skin and internal organs of systemic sclerosis, the differentiation of capillary wall-resident endothelial cells and pericytes into profibrotic myofibroblasts (i.e., endothelial-to-myofibroblast and pericyte-to-myofibroblast transitions) results in destructive vasculopathy characterized by progressive capillary rarefaction and contributes concomitantly to tissue fibrosis alongside fibroblast-to-myofibroblast transition.

On the contrary, EndoMT occurring in the thin wall of capillary vessels may result in an increase in the number of perivascular profibrotic myofibroblasts and a parallel loss of ECs, thus providing a unique link between tissue fibrosis and “destructive vasculopathy”, a process clinically evident in SSc skin by nailfold video-capillaroscopy and characterized by capillary rarefaction and disturbed angiogenic responses (Figure 3) [8,43,47]. Thus, the well-known capillary rarefaction commonly observed in fibrotic tissues should not be further regarded as a mere consequence of microvessel entrapment in the fibrotic ECM, but rather as an important pathogenic process fueling tissue fibrosis via EndoMT.

### 4.2. Pericytes

Pericytes are perivascular cells embedded in the basement membrane and directly interacting with the endothelium of capillaries, precapillary arterioles and postcapillary venules in all human organs [8,43,77,78]. Because of their close relationship with ECs, pericytes inside the capillary wall play an important role in endothelial barrier development/maintenance and their dysfunction or loss has been related to many microvascular diseases, including hypoxia, hypertension and diabetic retinopathy, as well as fibrosis, inflammation and cancer [79,80]. From a functional point of view, these perivascular cells display contractile properties with which they regulate blood flow by varying the microvessel diameter in response to vasoactive molecules [8,43,77], while immunophenotypically they are characterized by the expression of platelet-derived growth factor receptor-β (PDGFR-β), chondroitin sulphate proteoglycan 4 (also known as nerve/glial antigen 2 or NG2), CD135, nestin, desmin and the transcription factor FoxD1 [8,43,78]. Interestingly, they have also been shown to express the myofibroblastic marker α-SMA, an immunophenotypic feature that makes them an additional plausible source of activated myofibroblasts [8,43,78]. In this regard, several studies have indicated the evidence of a pericyte-to-myofibroblast transition in different fibrotic disorders including pulmonary, liver, kidney and myocardial fibrosis [81,82,83,84]. In addition, it has been recently demonstrated that pericyte-to-myofibroblast differentiation represents a primary hallmark of tissue fibrosis occurring during organ aging, with age-associated tissue-specific molecular changes in the endothelium driving the acquisition of a profibrotic phenotype in pericytes with progressive microvascular loss and tissue accumulation of myofibroblasts [84]. Of note, in skin- and muscle-wounding experiments, a disintegrin and metalloproteinase (ADAM)12+ lineage-derived pericytes were demonstrated to be able to migrate into the perivascular tissue and differentiate into myofibroblasts [85] and, in a similar way, pericytes were found to detach from the microvasculature, migrate into the interstitial space and express profibrotic proteins after spinal cord injury [86,87]. Likewise, pulmonary pericytes explanted from patients with idiopathic pulmonary fibrosis were reported to be proner to migrate and invade the surrounding extracellular matrix, suggesting that these cells may play an important role in the development of lung fibrosis [88].

As far as SSc is concerned, different preclinical and experimental studies underlined the contribution of pericytes to the appearance of profibrotic myofibroblasts [89,90,91,92,93]. In particular, in the affected skin of SSc patients with the diffuse cutaneous subset pericytes were proved to express type I collagen and the ED-A fibronectin splice variant, an isoform that is de novo expressed during wound healing and fibrotic changes [89], while in mouse pulmonary pericytes stimulation with TGF-β1 was able to increase mRNA expression of type I collagen, connective tissue growth factor (CTGF) and α-SMA, suggesting the occurrence of a transdifferentiation process in these cells [92]. Moreover, an immunophenotypic analysis of myofibroblasts and perivascular mesenchymal cells in the bleomycin-induced rat scleroderma model demonstrated that pericytes may be considered as possible myofibroblast progenitors in the sclerotic lesions of these animals [93].

Interestingly, since pericytes exhibit a great plasticity with an ability to differentiate into different mesenchymal populations such as osteoblasts, chondrocytes, adipocytes and fibroblasts, they can be considered a type of mesenchymal stromal/stem cells (MSCs) [8,43,94,95]. In addition, pericytes and MSCs derived from bone marrow (BM-MSCs) share some cell markers including PDGFR-β, α-SMA, NG2 and desmin, with BM-MSCs distributing around vessels and behaving as pericytes in assisting ECs to form and maintain a vascular network in a variety of experimental conditions [8,43,77,95]. Based on this evidence, recent studies employing SSc BM-MSCs as pericyte surrogates demonstrated that these stem cells display a myofibroblast-like phenotype and may participate in the accumulation of profibrotic myofibroblasts in SSc skin [96,97]. Moreover, it was demonstrated that SSc BM-MSCs express higher levels of ADAM12 in respect to healthy MSCs and that a pathologic microenvironment enriched in TGF-β1 may contribute to pericyte-to-myofibroblast differentiation by further increasing ADAM12 expression, which indeed acts as a positive regulator of the profibrotic TGF-β1 signaling cascade [97]. Furthermore, prolonged exposure of BM-MSCs to a profibrotic milieu mimicking the SSc microenvironment increased the tendency of these cells to differentiate into myofibroblasts [98]. Finally, similarly to human SSc BM-MSCs, it has been reported that also BM-MSCs from the KLF5+/−; Fli1+/− murine model of SSc display enhanced migration, proliferation and collagen production in response to TGF-β1, thus showing a preferential differentiation toward myofibroblasts instead of behaving as pericyte precursors [59].

Collectively, the aforementioned studies support the notion that, in concert with EndoMT, pericyte-to-myofibroblast transition inside the wall of capillary vessels may contribute to SSc-related “destructive vasculopathy” and the concomitant tissue accumulation of profibrotic myofibroblasts driving fibrosis of the skin and internal organs (Figure 3).

### 4.3. Vascular Smooth Muscle Cells

VSMCs are highly specialized cells that are located in the vascular tunica media and whose main functions consist of regulating blood vessel tone, stream and pressure [99]. While fully differentiated VSMCs exhibit a quiescent “contractile” phenotype, with a very low proliferation rate and the expression of specific contractile proteins (i.e., smooth muscle myosin heavy chain, smooth muscle 22α and calponin), after vascular injury they switch to a dedifferentiated or highly “synthetic” phenotype, becoming proliferative/migratory and displaying production of ECM proteins and reduced expression of VSMC-specific markers [99]. Such a “synthetic” phenotype has been found to participate in the development of different pathologic conditions including atherosclerosis, hypertension, and neointima formation [99,100,101,102]. Of note, VSMCs can also switch to the non-canonical myofibroblast-like phenotype, characterized by increased expression of fibronectin 1, osteoprotegerin, type I collagen and upregulation of fibroblast specific pathways [102,103]. These myofibroblast-like cells have been identified in mouse plaques, particularly accumulating in the fibrous cap, and in a single-cell RNA sequencing dataset from human coronary plaques [102,104], but such a phenotypic change in VSMCs might occur in the vessels of SSc patients, as well.

In this context, to date it has been demonstrated that SSc VSMCs are characterized by higher proliferation rates, increased metabolic activity and enhanced resistance to apoptosis, likely contributing to the altered structure of the vascular wall in SSc patients [105]. Moreover, when stimulated with agonistic anti-PDGFR autoantibodies isolated from SSc sera, healthy pulmonary VSMCs were shown to acquire a “synthetic” phenotype consisting of higher proliferation and migration activities, type I collagen production and reduced expression of the typical “contractile” markers, thus contributing to hyperplasia of the tunica media as well as intimal thickening [106].

It is well known that VSMCs may originate from multipotent BM-MSCs [107,108]. For this reason, a recent study analyzed the differentiation potential of SSc BM-MSCs toward VSMCs and fibroblasts in response to different key factors including CTGF, basic fibroblast growth factor (b-FGF) and TGF-β1 [98]. Indeed, the SSc profibrotic microenvironment may lead to distinct differentiation processes in such multipotent cells ranging from VSMCs with “contractile” or “synthetic” phenotypes to two functionally different fibroblast populations, namely fibroblasts and activated myofibroblasts [98]. Interestingly, the authors demonstrated that SSc BM-MSCs responded to CTGF with impaired physiologic “contractile” VSMC differentiation, and to b-FGF with a shift toward a “synthetic” phenotype. Mostly, SSc BM-MSCs exhibited increased commitment toward myofibroblast differentiation after induction with TGF-β1 in respect to healthy cells, with SSc-MSC-derived myofibroblasts functionally resembling activated SSc lesional fibroblasts [98]. Thus, the authors concluded that deregulated plasticity of VSMC progenitors might additionally contribute to the severity of SSc vasculopathy [98].

Altogether, currently available data indicate that a switch of VSMCs from a “contractile” to a “synthetic” and “proliferative” phenotype is one of the pathogenic mechanisms underlying the formation of SSc-related fibroproliferative vascular lesions (Figure 2).

## 5. Main Molecular Pathways Driving Myofibroblast Differentiation from Vascular Wall Residing Cells in Systemic Sclerosis and Related Therapeutic Strategies

Different molecular mechanisms and signaling pathways that underlie fibroblast-to-myofibroblast transition have been found to regulate also EndoMT, and are supposed to be involved in pericyte-to-myofibroblast transition and pathologic activation of VSMCs, as well [8,43,109,110,111,112]. Accordingly, several compounds have been tested or are currently under investigation in order to unravel their possible therapeutic efficacy in targeting the mechanisms underlying the differentiation of profibrotic myofibroblasts inside the vessel wall, likely ameliorating both the “fibroproliferative vasculopathy” and the “destructive vasculopathy” contributing to tissue fibrosis in SSc [113,114,115].

### 5.1. TGF-β Signaling

TGF-β, the main cytokine involved in the induction and maintenance of pathogenic fibrosis in SSc, may signal through two different signaling cascades, namely the canonical (Smad-dependent) and the non-canonical (Smad-independent) pathways. The canonical pathway takes place after TGF-β binding to type II receptor (TGF-βRII), which dimerizes and phosphorylates type I receptor (TGF-βRI), leading to its activation. Once activated, TGFβRI is able to phosphorylate Smad2 and Smad3 proteins, which form a complex with Smad4 and subsequently transfer to the nucleus, where they regulate the transcription of profibrotic genes. In the non-canonical pathway, activated TGF-βRs signal through different cascades including mitogen-activated protein kinase (MAPK) pathways, PI3K/AKT, Rho/Rac, Janus kinase/signal transducers and activators of transcription (JAK/STAT), as well as NF-κB, FAK, Src, c-Abl and PKC-δ, which regulate gene expression directly or together with Smad proteins [3,43,116]. In particular, activation of Smad-independent TGF-β cascade leads to the phosphorylation and consequent inhibition of glycogen synthase kinase-3β (GSK-3β) by means of the PKC-δ and c-Abl non-receptor kinases, with the following stabilization and nuclear translocation of Snail1. Once accumulated in the nucleus, Snail-1 determines the upregulation of mesenchymal markers and the parallel downregulation of endothelial markers, thus triggering the acquisition of a myofibroblast phenotype in ECs [3,43,116]. Given the pivotal role played by TGF-β in driving fibrosis, the inhibition of its signaling cascade may represent a potential antifibrotic therapeutic approach in different pathologic conditions, including SSc [117,118,119].

In this context, among the molecules capable of targeting TGF-β, it is worth mentioning the humanized anti-TGF-β antibody fresolimumab, which is able to bind and neutralize all three TGF-β isoforms and whose efficacy in reducing TGF-β-mediated gene expression in SSc skin has been described in a small single center study [120]. A significant antifibrotic effect was also reported for the antidiabetic agent metformin which was demonstrated to inhibit TGF-β1-induced fibrosis and myofibroblast differentiation in different fibrotic models including experimental scleroderma [121], and the two tyrosine kinase inhibitors nilotinib and imatinib, which are both able to target PDGFR and c-Abl, the latter being one of the key non-receptor tyrosine kinases mediating not only the non-canonical TGF-β pathway, but also VEGFR and FGFR signaling [43,117,122]. In particular, both nilotinib and imatinib have been shown to prevent TGF-β- and PDGF-induced collagen synthesis in skin fibroblasts, as well as to decrease myofibroblast formation in experimental scleroderma [43,117,122]. However, only imatinib was reported to inhibit EndoMT in murine models of PAH induced by hypoxia [123,124]. Of note, successive trials on these tyrosine kinase inhibitors failed to prove their clinical efficacy in SSc patients [43,117]. Another potent tyrosine kinase inhibitor representing a potential SSc disease-modifying agent is nintedanib, which is capable of simultaneously targeting PDGFR, FGFR, VEGFR and several non-receptor tyrosine kinases including c-Abl, Src, MAPK and extracellular signal-regulated kinase (ERK) [122]. Indeed, this compound has been demonstrated to significantly inhibit SSc fibroblast-to-myofibroblast differentiation, and to lower myofibroblast count and skin fibrosis in different preclinical models of SSc [125]. Furthermore, nintedanib strongly reduced lung fibrosis in the intratracheal bleomycin- or silica-induced mouse models and inhibited TGF-β2-induced collagen production in healthy human pulmonary fibroblasts [126]. Of note, nintedanib was also shown to ameliorate experimental PAH via inhibition of EndoMT and pulmonary arterial VSMC proliferation, suggesting that this molecule might be used as a therapeutic agent for PAH by preventing fibroproliferative vascular remodeling [127]. Clinically, treatment with nintedanib was able to reduce the decline in forced vital capacity in SSc patients suffering from ILD [128,129,130], even if when considering patients with a background therapy with mycophenolate, such a decline did not significantly differ from placebo-treated patients [131]. In addition, given its relevant side effects, the effective safety and efficacy of nintedanib for SSc treatment has yet to be fully investigated [43,117]. Finally, the third-generation kinase inhibitor bosutinib, able to simultaneously inhibit c-Abl and Src, was reported to reduce type I collagen, fibronectin and α-SMA production in SSc dermal fibroblasts in vitro [132] and to exert antifibrotic effects in vivo, with a significant decrease in myofibroblast activation and profibrotic gene expression in TBRIcaCol1a2Cre-transgenic mice [133].

As previously mentioned, during Smad-independent TGF-β signaling cascade, c-Abl acts together with PKC-δ to phosphorylate and inhibit GSK-3β, thus leading to Snail1 activation and consequent mesenchymal marker upregulation. In the absence of GSK-3β phosphorylation, GSK-3β is active and induces proteasomal Snail1 degradation, finally resulting in the abrogation of EC transition into a mesenchymal/myofibroblast phenotype [8,43,122]. In this context, blockade of GSK-3β phosphorylation by specific inhibition of PKC-δ with rottlerin effectively prevented the TGF-β1-induced EndoMT program in murine pulmonary ECs [134].

Given the capability of TGF-β to promote the activation of PI3K/mTOR pathway [117], it has been demonstrated that the inhibition of mTOR complex activity by rapamycin was able to prevent EndoMT in vitro and ex vivo [135,136] and to improve fibrosis in two different mouse models of SSc [137]. Clinically, treatment with rapamycin was shown to improve skin stiffness, with good tolerability, in a small number of SSc patients [138,139,140]. Of note, the direct contribution of rapamycin in preventing SSc-related EndoMT has yet to be clarified, even in light of the recent conflicting results demonstrating the capability of this compound in promoting EndoMT in human coronary artery ECs undergoing stress-induced premature senescence [141]. Other molecules able to interfere with mTOR signaling, thus playing antifibrotic effects, are represented by the iridoid glycoside geniposide and the phytochemical drug tanshinone IIA, both reported to inhibit EndoMT in the bleomycin-induced scleroderma mouse model [142,143]. Interestingly, tanshinone IIA was also shown to exert an inhibitory effect on IL-17A-induced SSc dermal VMSC proliferation, collagen synthesis and migration [144].

Other molecules able to interfere with TGF-β signaling that have been reported to block EndoMT in SSc include the inhibitor of the dipeptidyl peptidase-4 linagliptin [145,146] the major active constituent of licorice root glycyrrhizin, which ameliorated fibrosis, vasculopathy and inflammation in SSc animal models [147], and AdipoRon, a novel small molecule selective for adiponectin receptors that improved bleomycin-induced dermal fibrosis in mice by attenuating both fibroblast proliferation and EndoMT [148].

In addition, even if not directly investigated in SSc, hepatocyte growth factor and the aldosterone receptor-blocker spironolactone were found to prevent TGF-β1-induced EndoMT in human umbilical vein ECs [119,149,150]. Finally, TGF-β1-induced EndoMT was reverted after treatment with triazole arginylglycylaspartic acid antagonist in endothelial precursor cells [151].

### 5.2. Developmental Pathways

Wnt/β-catenin, also known as canonical Wnt signaling, Notch, Hedgehog (Hh) and Hippo are all developmental signaling pathways mutually interconnected and orchestrated by TGF-β that are dysregulated in SSc and may contribute to myofibroblast accumulation and fibrosis [152,153,154].

The Wnt/β-catenin signaling cascade starts with the binding of Wnt ligands to their cell surface Frizzled receptors and low-density lipoprotein co-receptors, leading to the degradation of the multiprotein β-catenin destruction complex and finally resulting in β-catenin nuclear translocation and consequent target gene transcription [155,156]. Pharmacological inhibition of β-catenin with PKF118-310 and ICG-001 was shown to prevent and reverse dermal fibrosis by ameliorating skin thickness and reducing myofibroblast counts ex vivo [157], while in SSc patients, local treatment of affected skin with the β-catenin inhibitor C82 reduced a specific cluster of genes known to be associated with SSc but did not ameliorate the modified Rodnan skin score (mRSS) [158]. In addition, in light of the recently demonstrated crosstalk between the adenosine A2a receptor (A2aR) and Wnt/β-catenin pathway that may promote collagen production, pharmacological blockade of A2aR with the selective antagonist istradefylline was shown to reduce myofibroblast accumulation in the murine model of bleomycin-induced dermal fibrosis [159]. Finally, in light of the overexpression of X-linked inhibitor of apoptosis protein (XIAP) in SSc skin, its inhibition has been investigated in two different murine models of SSc [160]. In particular, XIAP was demonstrated to amplify the profibrotic effects of Wnt/β-catenin signaling, and its inactivation by small interfering RNA (siRNA)-mediated knockdown and pharmacological inhibition was shown to block Wnt/β-catenin-dependent transcription and consequent aberrant fibroblast activation [160].

The Notch signaling cascade consists of membrane-bound ligands known as Delta-like (Dll1, Dll3, Dll4), Jagged1 and Jagged2, and Notch receptors (referred to as Notch1, Notch2, Notch3, Notch4) that can interact within the same cell (cis) or across cell boundaries (trans) [152,161,162]. The binding between Notch receptors and ligands results in γ-secretase-mediated Notch cleavage and consequent release of the active Notch intracellular domain (NICD) that, once translocated into the nucleus, aggregates with the CSL transcription factor complex and stimulates the transcription of Notch target genes [152,161,162]. Several studies demonstrated the activation of Notch signaling in SSc skin and fibroblasts, as well as in SSc mouse models [152,163,164,165], but only one investigated pharmacological inhibition of such pathway in this pathologic condition [165]. In particular, in this study, the authors showed that Notch blockade through the overexpression of a Notch anti-sense structure or by the γ-secretase inhibitor DAPT could prevent fibroblast-to-myofibroblast differentiation in two different mouse models of SSc [165]. Although different studies reported that Notch signaling pathway might participate in EndoMT induction, contributing to neointimal hyperplasia [166,167,168], and that the already mentioned rapamycin may function as an effective EndoMT inhibitor by suppressing also Notch activation [169], no data about a direct implication of Notch in SSc-related EndoMT are currently available.

The Hh pathway is regulated by three different proteins, namely Hh ligand, Patched (Ptch) receptor and Smoothened (Smo). The signaling cascade starts when Hh ligand binds and inactivates Ptch receptor, with the Hh–Ptch complex being internalized and degraded by the proteasome. Ptch normally inhibits Smo, a protein that is located within the cell inside a vesicle, but when Ptch is degraded, Smo is free to migrate from the vesicle and embed in the membrane. This leads to the activation of an important pool of proteins, called Gli proteins, which move into the nucleus and activate the transcription of Hh target genes [170,171]. As far as therapy against Hh signaling, targeting of Smo either genetically by siRNA or pharmacologically via the small molecule inhibitor LDE223 in two SSc mouse models was reported not only to prevent dermal thickening, myofibroblast differentiation and accumulation of collagen, but also to regress established fibrosis [172]. Moreover, the synthetic molecule pirfenidone, recently approved for the treatment of idiopathic pulmonary fibrosis [173], was demonstrated to inhibit lung fibrosis by blocking both Hh [174,175] and TGF-β signaling [176,177]. Nevertheless, in a randomized clinical trial, therapeutic use of pirfenidone in SSc patients with ILD had no beneficial effects on disease outcomes [178]. Finally, beyond treating each of these developmental signaling pathways individually, simultaneous targeting of Wnt, Notch and Hh demonstrated antifibrotic effects in the bleomycin-induced mouse model and in mice overexpressing a constitutively active TGF-βRI [179].

The Hippo pathway is triggered by different stimuli and consists of a core kinase cascade in which MST1 and MST2 kinases bind to Salvador (SAV)/WW45, forming a complex that phosphorylates and activates the MOB1A/B subunits of LATS1/2 kinases. In turn, activated LATS1/2 phosphorylate and inhibit the transcriptional co-activators YAP and TAZ, resulting in their cytoplasmic retention and degradation. Conversely, when dephosphorylated, YAP and TAZ translocate into the nucleus and activate gene transcription [180,181]. As YAP and TAZ are key matrix stiffness-regulated coordinators of fibroblast activation and matrix synthesis, therapeutic blockade of the Hippo pathway has been investigated in SSc. In particular, therapeutic targeting of TAZ and YAP by dimethyl fumarate was reported to be effective in preventing bleomycin-induced fibrosis in mice and in blocking the pro-fibrotic effects of TGF-β in cultured SSc skin fibroblasts [182]. Moreover, selective YAP/TAZ inhibition via dopamine receptor D1 agonism was found to reduce pro-fibrotic gene expression in fibroblasts of mice treated with intratracheal bleomycin [183].

### 5.3. Janus Kinase/Signal Transducers and Activators of Transcription (JAK/STAT) Signaling Pathway

JAK/STAT pathway begins with the autophosphorylation/activation of the receptor-associated tyrosine kinases JAK by binding a ligand (growth factors, interferon or cytokines) to its cognate transmembrane receptor. JAK then recruits and phosphorylate/activate STAT proteins, which translocate into the nucleus and promote the transcription of target genes [184,185,186]. Increasing evidence suggests prominent JAK/STAT activation in cultured SSc fibroblasts, in SSc dermal and lung biopsies and in experimental scleroderma [187,188,189]. In particular, it has been demonstrated that the hyperactivation of STAT3, which may be induced by various cytokines such as IL-6 and oncostatin M, makes SSc fibroblasts more prone to differentiate into myofibroblasts [116,187,190]. Regarding the therapeutic targeting of the JAK/STAT signaling pathway, pharmacological inhibition of STAT3 was reported to prevent TGF-β-mediated myofibroblast formation in both experimental skin and lung fibrosis [43,116], while the selective JAK-2 blockade by the specific inhibitor TG101209 or by siRNA significantly reduced collagen synthesis in SSc fibroblasts and prevented fibrosis in two different SSc mouse models [189]. In addition, JAK-1 inhibition with ruxolitinib was shown to reduce cytokine-mediated collagen production in dermal fibroblasts [191], whereas targeting JAK signaling with tofacitinib demonstrated potent antifibrotic effects in different SSc mouse models [184]. In light of such promising results, a phase I/II study of tofacitinib in subjects with diffuse cutaneous SSc is currently in progress [117,118,185].

### 5.4. Additional Therapeutic Targets

ET-1 is known to be a key regulator of vasoconstriction and fibrosis in SSc [9,192]. In particular, ET-1 has been found to contribute to the EndoMT process through a synergistic stimulation of the Smad-dependent TGF-β pathway [8]. In this context, ET-1 receptor antagonism with bosentan and macitentan was effective in inhibiting EndoMT in SSc both in vitro and ex vivo [53,54,193,194].

As already mentioned, in SSc, MMP-12-dependent cleavage of uPAR is implicated in inducing the EndoMT process [8,46]. Indeed, by determining an increase in integrin-dependent cell adhesion to the ECM and generating the cell tension necessary for the assembly of α-SMA into stress fibers, uPAR cleavage results in the acquisition of a myofibroblast-like phenotype [8]. Interestingly, specific inhibition of MMP-12 with MMP408 significantly dampened the profibrotic effects of SSc sera on the expression of genes related to EndoMT in healthy microvascular ECs [46].

Other profibrotic orchestrators implicated in SSc that have been investigated as potential therapeutic targets, even if not specifically for the inhibition of EndoMT, include oncostatin M, CTGF, IL-6, IL-1β and the chemokine c-c motif ligand 24 (CCL24) [43,117]. The inhibition of oncostatin M in SSc patients is now under evaluation in a phase II clinical trial [43,117], while the blockade of CTGF with a monoclonal antibody was shown to ameliorate skin fibrosis in SSc mouse models and to retard the decline in forced vital capacity in patients with idiopathic pulmonary fibrosis [43,117,195]. Interestingly, since the small inhibitor of Rho/myocardin-related transcription factor (MRTF)/serum response factor (SRF)-mediated gene transcription CCG-203971 was able to inhibit CTGF, α-SMA, and collagen synthesis in both SSc fibroblasts and experimental scleroderma [196], novel inhibitors of the Rho/MRTF/SRF transcription pathway are now under investigation as potential antifibrotic drugs for SSc treatment [197,198].

As far as IL-6 is concerned, treatment of dermal SSc fibroblasts with tocilizumab, a humanized antibody directed against IL-6 receptor, downregulated the expression of different pro-fibrotic genes and reduced fibroblast migration and contractility [199]. In SSc patients, tocilizumab demonstrated encouraging protective effects on skin thickening and loss of forced vital capacity in a phase II trial [200,201], and preserved lung function in patients with early SSc-ILD without ameliorating the clinical parameter of skin fibrosis extent mRSS in a recent phase III trial [202].

Rilonacept, also known as “IL-1 Trap”, is a fusion protein that binds and neutralizes IL-1β. On these bases, this molecule was tested in a phase I/II trial performed in SSc patients, but unfortunately it had no significant clinical effect [203].

CCL24 blockade with the monoclonal antibody CM-101 was reported to decrease SSc-serum induced dermal fibroblast-to-myofibroblast differentiation in vitro and to prevent experimental dermal and pulmonary fibrosis [204].

Peroxisome proliferator-activated receptor (PPAR)-γ is a molecule with antifibrotic properties that is downregulated in SSc skin and whose pharmacological activation with its agonist rosiglitazone has been demonstrated to ameliorate fibrosis in mice [118]. Moreover, the pan-PPAR agonist IVA337 was reported to be effective in mitigating fibrosis in preclinical models of SSc [205,206], even if in a phase IIb clinical trial performed on diffuse cutaneous SSc patients it failed to improve mRSS [114].

The synthetic retinoid tamibarotene (Am80) has been reported to significantly attenuate dermal/hypodermal fibrosis and EndoMT in bleomycin-treated mice [207], while the synthetic analogue of prostacyclin iloprost was able to promote angiogenesis and prevent EndoMT in SSc microvascular ECs [208].

Recently, it has been demonstrated that the monoterpene glycoside paeoniflorin, a molecule with endothelial protection and antifibrotic properties, significantly prevented chronic hypoxia/SU5416-induced PAH in rats, with a therapeutic effect likely being related to its capability to inhibit EndoMT in lung arterial ECs [209].

Another drug with potential antifibrotic properties that already proved efficacy in patients with PAH is represented by the soluble guanylate cyclase stimulator riociguat [210]. The effects of this compound on skin involvement were recently investigated in early diffuse cutaneous SSc patients in a phase II study and, although the primary endpoint consisting of changes in mRSS was not reached, secondary endpoints regarding differences in mRSS progression rate suggested a positive effect for SSc patients [211].

Other molecules that are under investigation for their antifibrotic effects are cannabinoids, pleiotropic arachidonic acid-derived small molecules that signal through CB1 and CB2 G-protein-coupled transmembrane receptors [43,117]. Since SSc fibroblasts overexpress both CB1 and CB2 receptors, when incubated with the synthetic cannabinoid Win55,212–2, these cells were shown to be less prone to undergo fibroblast-to-myofibroblast differentiation [212]. Treatment with Win55,212–2 also ameliorated experimental bleomycin-induced skin fibrosis, whereas the CB2 receptor agonist JWH133 was shown to reduce experimental pulmonary fibrosis [213,214]. In light of these promising results, the synthetic CB2 receptor agonist lenabasum, which effectively reduced mRSS in a phase II study performed on SSc patients [215], is currently being tested in a phase III trial [117,118].

Finally, a recent study demonstrated that transmembrane receptor CD248 is significantly overexpressed in SSc perivascular cells and likely involved in pericyte-to-myofibroblast transition [216]. Strikingly, its blockade on SSc BM-MSCs employed as pericyte surrogates strongly prevented the differentiation of such cells toward myofibroblasts, thus paving the way for possible novel therapeutic strategies able to counteract the acquisition of a myofibroblast phenotype by pericytes in SSc [216].

**Figure 4:**
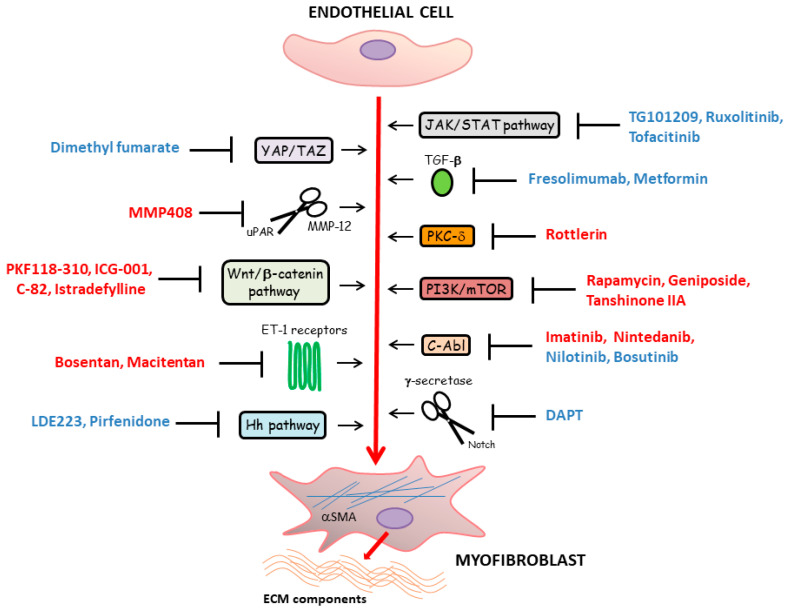
In systemic sclerosis, different signaling pathways underlying fibroblast-to-myofibroblast transition have been found to also regulate endothelial-to-myofibroblast transition [8,43,109,110,111,112]. The compounds targeting endothelial cell differentiation toward profibrotic myofibroblasts that have been tested or are currently under investigation are represented in red font. Other therapeutic molecules that demonstrated promising results in counteracting tissue fibrosis and that might have therapeutic efficacy in fighting also endothelial-to-myofibroblast transition are represented in blue font.

The main compounds targeting the EndoMT process and other therapeutic molecules demonstrating promising results in counteracting SSc-related tissue fibrosis and potentially interfering with EndoMT are represented in Figure 4.

## 6. Conclusions

As described in this review, microvascular disease represents a prominent pathologic event in SSc, with vascular wall-resident cells including ECs, pericytes and VSMCs likely being the initial target of the disease process. In particular, increasing evidence reported that, once injured, ECs may undergo two different fates: cell activation, finally resulting in impaired angiogenesis, tissue inflammation and luminal narrowing, or apoptosis, accounting for the loss of peripheral capillary network and consequent tissue ischemia. In addition, the recently described EndoMT process, during which ECs switch toward profibrotic myofibroblasts, is known to strongly contribute to both SSc-related fibroproliferative vascular lesions and capillary rarefaction accompanying the development of progressive tissue fibrosis. Of note, pericyte-to-myofibroblast differentiation and VSMC acquisition of a myofibroblast-like “synthetic” phenotype may additionally participate in these crucial pathologic events. In recent years, the principal mechanisms underlying the formation and consequent accumulation of profibrotic myofibroblasts in SSc have been investigated in depth, leading to the discovery of different molecular pathways (i.e., TGF-β and ET-1 cascade, developmental pathways and JAK/STAT signaling), against which several antifibrotic agents have been tested in preclinical models or are currently under evaluation in different clinical trials. At present, no effective curative strategies are available for SSc treatment, and the main therapeutic approach is merely symptomatic. Thus, the identification of novel drugs able to prevent vascular injury and consequent tissue fibrosis, together with a deeper understanding of preferential associations of different microvascular alterations with specific SSc subsets, may provide the rationale for counteracting the most significant pathogenic aspects and developing more effective and personalized therapies for SSc patients.
